# Psychobehavioral profiles of eating risk: schema–emotion regulation patterns differ in central adiposity with genotype (FTO rs9939609) as external validator—a pilot study

**DOI:** 10.1007/s40519-026-01853-5

**Published:** 2026-04-29

**Authors:** Małgorzata Obara-Gołębiowska

**Affiliations:** Department of Clinical, Developmental and Educational Psychology, Faculty of Social Sciences, University of Warmia and Mazury in Olsztyn, Ul. Dybowskiego 13, 10-723 Olsztyn, Poland

**Keywords:** Early maladaptive schemas, Emotion regulation, Eating behavior, Cluster analysis, Central adiposity, Health education

## Abstract

**Purpose:**

To identify person-centered psychological profiles of eating-related risk based on early maladaptive schemas (EMS), emotion regulation difficulties, and eating behaviors, and to examine their external validation using anthropometric indices (BMI, waist circumference, WC) and the FTO rs9939609 genotype.

**Methods:**

Fifty women aged 18–35 years (approximately balanced between normal weight and overweight/obese groups) completed the YSQ-S3, DERS-36, and QERB. Standardized variables were clustered with k-means (*k* = 2–4); the optimal solution was selected via silhouette and BIC with stability checks. BMI and WC were used for external validation; FTO rs9939609 (AA/AT/TT) was examined using χ^2^/Fisher’s tests.

**Results:**

A two-cluster solution best fit the data. Cluster 1 (*n* = 23) showed higher EMS, greater emotion dysregulation, and higher emotional/habitual overeating, alongside higher restraint, whereas Cluster 2 (*n* = 27) showed a consistently lower-risk profile. Cluster 1 presented higher WC (*M* = 92.74 cm, SD = 12.86) and BMI (*M* = 30.44, SD = 5.18) than Cluster 2 (WC: *M* = 75.19 cm, SD = 18.60; BMI: *M* = 22.65, SD = 4.15); differences were significant in parametric and nonparametric tests (all *p* < 0.001; Hedges’ *g* = 1.07–1.65). The FTO genotype distribution did not differ between clusters (*χ*^2^(2) = 0.33, *p* = 0.848).

**Conclusions:**

Distinct schema–emotion regulation profiles may be observable in young women and appear to align with central adiposity and BMI. While FTO rs9939609 did not differentiate clusters, person-centered profiling may help inform hypothesis generation regarding schema- and regulation-focused strategies in weight-related risk, with potential relevance for both clinical and educational contexts.

**Level of evidence:**

Level III, observational analytic (cross-sectional) study.

**Supplementary Information:**

The online version contains supplementary material available at 10.1007/s40519-026-01853-5.

## Introduction

Obesity and overweight are complex health conditions shaped by the interplay of biological, psychological, and behavioral mechanisms [1, 2]. Genetic variation, such as FTO polymorphisms, confers susceptibility to excess weight, but research increasingly highlights proximal determinants—including early maladaptive schemas (EMS) [3, 4], difficulties in emotion regulation [5, 6], and disordered eating behaviors [7] as central to the development and persistence of unhealthy eating patterns. These processes often co-occur, creating self-reinforcing cycles of dysregulation that increase vulnerability to weight gain and central adiposity.

Evidence indicates that individuals with obesity frequently present elevated schemas from the Disconnection and Rejection and Impaired Limits domains [8–10], which intensify feelings of insecurity or inadequacy and promote maladaptive coping, such as emotional eating. Emotion dysregulation, particularly poor impulse control and limited access to adaptive strategies, is common among individuals with higher BMI [10, 11], and can mediate the relationship between schemas and disordered eating [12]. In turn, eating behaviors such as emotional eating, habitual eating, and dietary restraint both reflect and reinforce these vulnerabilities, contributing to the maintenance of excess weight [13–15].

Most prior studies have examined EMS, emotion regulation, and disordered eating in isolation. Variable-centered approaches capture associations between these constructs but may overlook subgroups of individuals who share distinct risk constellations. Cluster-based methods offer an alternative, person-centered perspective, capable of identifying clinically meaningful psychological profiles that cut across diagnostic categories [16]. Such profiles are particularly relevant in eating behavior research, where maladaptive schemas, emotion dysregulation, and eating patterns often cluster in characteristic ways.

Health risks linked to these psychological patterns extend beyond BMI to central adiposity, a stronger marker of cardiometabolic disease [17]. Importantly, diet quality, such as consumption of ultra-processed foods, also elevates health risks independently of body weight [18]. Identifying psychological profiles associated with greater waist circumference may, therefore, illuminate mechanisms by which cognitive and emotional vulnerabilities translate into adverse health outcomes.

External validation enhances the interpretability of psychological profiling. The FTO rs9939609 polymorphism, which has been consistently associated with adiposity and eating behavior [2], provides a biologically grounded marker to test whether identified psychological subgroups correspond to differential genetic distributions. While genetic effects are modest and often mediated by psychological and behavioral pathways, their inclusion as external validators strengthens the ecological validity of person-centered findings.

This pilot study is part of a broader research program aimed at developing an integrative model of psychological, behavioral, and genetic determinants of eating and weight regulation [19]. While other analyses within this program have focused on variable-centered associations, the present work adopts a person-centered approach to identify clusters defined by schemas, emotion regulation, and disordered eating behaviors. By examining differences in BMI and waist circumference, and by testing FTO distribution as an external validator, this study provides complementary insights that advance the overarching framework.

Building on prior evidence, the present pilot study adopted a person-centered approach to examine whether distinct psychological profiles of eating-related risk, defined by early maladaptive schemas, emotion regulation difficulties, and disordered eating behaviors, can be empirically identified. It was further expected that individuals characterized by a higher-risk psychological configuration would present with higher anthropometric indicators (BMI and waist circumference) compared to those with a lower-risk profile. Given previous research linking FTO rs9939609 to obesity-related phenotypes, genotype distribution was additionally explored as an external biological validator of the identified clusters. Due to the pilot nature of the study, these analyses were treated as exploratory and hypothesis-generating.

## Materials and methods

### Participants

The study included 50 women aged 18–35 years. Participants were recruited between March and September 2020 through announcements distributed within the university community (University of Warmia and Mazury in Olsztyn). Data were collected in person in a laboratory setting by trained research staff.

Eligibility criteria included female sex, age between 18 and 35 years, current university enrollment, and BMI within the normal-weight or overweight/obese range. Exclusion criteria comprised pregnancy, lactation, legal incapacity, refusal to provide informed consent, intellectual disability, severe psychiatric disorders, substance dependence, and current psychiatric treatment (e.g., schizophrenia or bipolar disorder).

The sample was balanced by design into a normal-weight group (BMI 18.5–24.9 kg/m^2^; *n* = 25) and an overweight/obese group (BMI ≥ 25 kg/m^2^; *n* = 25) for descriptive purposes. Group allocation by body weight status was not used in inferential analyses, which were based exclusively on clusters derived from psychological variables (YSQ domains, DERS, and QERB subscales).

All participants were university students, predominantly enrolled in bachelor’s or master’s degree programs.

Although the inclusion criteria targeted the normal-weight and overweight/obese BMI ranges, one participant presented a BMI slightly below the predefined lower threshold (BMI = 16.34). Given the minimal deviation and the exploratory pilot character of the study, this case was retained in the analyses.

Descriptive characteristics of the sample are presented in the Results section. No additional socioeconomic variables were collected in this pilot study.

### Anthropometric assessment

Measurements were obtained with participants wearing light clothing and no shoes. Body weight and height were measured by trained research personnel using calibrated medical equipment (SECA 515mBCA, Allers, Hamburg, Germany; Radwag WPT 100/200, Poland) following standard anthropometric procedures. Waist circumference (WC) was measured using a non-elastic tape positioned midway between the lowest rib and the iliac crest. BMI was calculated as weight (kg) divided by squared height (m^2^) and classified according to WHO criteria.

### Genetic analysis

Genotyping was performed for the FTO rs9939609 polymorphism. Buccal swab samples were collected from each participant using sterile collection kits**.** DNA extraction and genotyping were performed by a certified external laboratory (Genomed, Poland). The rs9939609 variant was genotyped using polymerase chain reaction (PCR) followed by sequencing. Based on genotyping results, participants were classified as AA, AT, or TT carriers. Genotype frequencies are reported in the Results section.

### Ethics

The study protocol was approved by the Ethics Committee for Scientific Research at the University of Warmia and Mazury in Olsztyn (Decision No. 3/2019). All participants provided written informed consent prior to participation, and procedures were conducted in accordance with the Declaration of Helsinki.

### Measures

#### Young schema questionnaire—short form 3 (YSQ-S3, Polish version)

The Young Schema Questionnaire—Short Form 3 (YSQ-S3-PL) [3, 20] comprises 90 items assessing 18 early maladaptive schemas grouped into five higher-order domains. Responses are rated on a 6-point Likert scale, with higher scores reflecting stronger schema endorsement. The Polish version demonstrates good reliability in prior studies (*α* = 0.71–0.93). In the present sample, internal consistency for the five schema domains was good (Cronbach’s *α* = 0.81).

#### Questionnaire of eating-related behaviors (QERB)

The Questionnaire of Eating-Related Behaviors (QERB) [21] consists of 30 dichotomous items (yes/no) covering Habitual Overeating (HO), Emotional Overeating (EO), and Restrained Eating (RE). Higher scores indicate stronger maladaptive eating tendencies. Internal consistency has been reported as high in prior research (*α* = 0.89). In the present study, analyses were conducted using subscale totals; therefore, internal consistency was not recalculated in the present sample. EO and HO were analyzed as primary indicators, with RE considered exploratorily.

#### Difficulties in emotion regulation scale-36 (DERS-36)

The Difficulties in Emotion Regulation Scale-36 item version (DERS-36) [6, 22] includes 36 items rated on a 5-point Likert scale across six domains of emotion dysregulation. Higher totals represent greater difficulties. The Polish version shows good reliability in previous research (*α* = 0.88). In the present study, analyses were based on the validated total score; therefore, internal consistency was not recalculated. For this study, the global score was used as a composite index.

### Statistical analysis

Analyses were conducted in Python 3.11 using pandas, NumPy, SciPy, and scikit-learn.

Prior to analysis, missing data were screened and found to be minimal; complete-case analysis was, therefore, applied. Psychological variables (YSQ domains, DERS total, and QERB subscales EO, HO, RE) were standardized to *z*-scores.

K-means clustering was selected as an appropriate person-centered method for this pilot sample due to the modest sample size and the exploratory aim of identifying preliminary psychological configurations. Models with *k* = 2–4 were estimated. The optimal solution was determined using silhouette coefficients, Bayesian Information Criterion (BIC), and stability checks based on repeated random starts and bootstrap resampling. For k-means, BIC was approximated from the within-cluster sum of squares under a spherical Gaussian assumption to enable comparison across k. Cluster stability was evaluated by examining the consistency of cluster assignments across bootstrap resamples (see Supplementary Table S2).

Anthropometric indices (BMI and WC) were intentionally excluded from cluster formation to preserve the psychological nature of the clustering solution and were used only in subsequent external validation analyses.

Cluster differences in BMI and WC were examined using ANOVA or Kruskal–Wallis tests when assumptions were violated. Pairwise effects were expressed as Cohen’s d or Hedges’ g with 95% confidence intervals. Differences in FTO genotype distribution between clusters were tested using *χ*^2^ or Fisher’s exact tests with Cramér’s V as the effect size.

Cluster centroids (z-scores for EMS, DERS, EO, HO, and RE) were visualized using radar and bar plots. Equivalence testing (TOST) was treated as a supplementary sensitivity analysis for null effects and is reported in the Supplementary Materials.

The significance threshold was set at *α* = 0.05 (two-tailed). Additional diagnostics (tests of normality, cluster fit indices, and sensitivity analyses of FTO coding) are provided in the Supplementary Materials (Tables S1–S3).

## Results

### Preliminary analyses

All psychological variables (five YSQ domains, DERS total, and QERB subscales EO, HO, RE) were standardized to z-scores prior to clustering. Assumptions for parametric testing were evaluated; several variables deviated from normality, and anthropometric indices showed non-normal distributions. The assumption of homogeneity of variance was generally met. Where assumptions were violated, corresponding nonparametric tests are reported alongside parametric results. Detailed diagnostics are provided in the Supplementary Materials (Table S1). No influential outliers were identified.

### Cluster solution

A two-cluster solution was identified as optimal based on silhouette coefficients and BIC, with stable replication across resampling procedures. Supplementary fit indices for alternative models (*k* = 2–4) are presented in the Supplementary Materials (Table S2).

Standardized cluster centroids are presented in Table 1. Cluster 1 was characterized by higher endorsement of early maladaptive schemas, greater emotion regulation difficulties, and elevated emotional and habitual overeating, together with higher restraint. In contrast, Cluster 2 displayed consistently lower psychological risk levels.

**Table 1:** Standardized cluster centroids (*z*-scores) for psychological variables

Variable	Cluster 1 (high risk)	Cluster 2 (low risk)
Disconnection/rejection	0.72	− 0.62
Impaired autonomy	0.77	− 0.65
Impaired limits	0.46	− 0.39
Other directedness	0.64	− 0.54
Overvigilance	0.48	− 0.41
DERS total	0.65	− 0.55
Emotional overeating	0.46	− 0.39
Habitual overeating	0.77	− 0.66
Restrained eating	0.62	− 0.53

Values are standardized z-scores. Positive scores indicate above-average endorsement within the total sample

### Anthropometric validation

#### Descriptive anthropometric characteristics of the sample are presented in Table 2.

**Table 2:** Sample characteristics (*N* = 50)

Variable	Statistic
Body weight status, *n* (%)	
– Normal weight	25 (50.0)
– Overweight/obese	25 (50.0)
Age, *M* (SD)	22.68 (4.63)
Age range	19–35
BMI, *M* (SD)	26.23 (6.04)
BMI range	16.34–41.58
WC (cm), *M* (SD)	81.52 (9.34)
WC range (cm)	68–99

*WC* waist circumference, *BMI *body mass index

Clusters differed significantly in waist circumference (WC) and body mass index (BMI). Participants in the higher-risk cluster (Cluster 1) had higher WC (*M* = 92.74 cm, SD = 12.86) and BMI (*M* = 30.44, SD = 5.18) compared to the lower-risk cluster (Cluster 2) (WC: *M* = 75.19 cm, SD = 18.60; BMI: *M* = 22.65, SD = 4.15).

Differences were statistically significant in both parametric (WC: *t*(43.6) = 3.93, *p* < 0.001; BMI: *t*(45.7) = 5.79, *p* < 0.001) and nonparametric tests (WC: *H* = 15.18, *p* < 0.001; BMI: *H* = 23.40, *p* < 0.001). Effect sizes were large (WC: Hedges’ *g* = 1.07; BMI: *g* = 1.65).

Importantly, BMI and WC were not included in cluster formation and were used exclusively as external validators. Detailed statistics are presented in Table 3.

**Table 3:** Anthropometric validation of clusters

Measure	Cluster 1 (*n* = 23)	Cluster 2 (*n* = 27)	Test statistic	*p* value	Effect size
WC (cm)	*M* = 92.74, SD = 12.86	*M* = 75.19, SD = 18.60	*t*(46.2) = 3.93; *H* = 15.18	< 0.001	Hedges’ *g* = 1.07
BMI	*M* = 30.44, SD = 5.18	*M* = 22.65, SD = 4.15	*t*(42.0) = 5.79; *H* = 23.40	< 0.001	Hedges’ *g* = 1.65

*WC* waist circumference, *BMI* body mass index. Both parametric (*t* test) and nonparametric (Kruskal–Wallis *H*) results are shown

### Genetic validation

The distribution of FTO rs9939609 genotypes by cluster is presented in Table 4.

**Table 4:** Distribution of FTO rs9939609 genotypes by cluster

Genotype	Cluster 1 (*n* = 23)	Cluster 2 (*n* = 27)
AA	7	7
AT	10	11
TT	6	9

No significant differences in genotype distribution between clusters

The distribution of FTO rs9939609 genotypes (AA, AT, TT) did not differ significantly between clusters, *χ*^2^(2) = 0.33, *p* = 0.848, Cramér’s *V* = 0.08. Sensitivity analyses using additive and dominant coding yielded consistent null results (Supplementary Table S3).

### Profile interpretation

Relative to the lower-risk cluster (Cluster 2), the higher-risk cluster (Cluster 1) showed higher scores across EMS (notably Disconnection/Rejection and Impaired Autonomy), greater overall emotion dysregulation (DERS), and elevated emotional and habitual overeating (EO/HO), alongside higher restraint (RE). This pattern was accompanied by substantially higher WC and BMI.

**Fig. 1:**
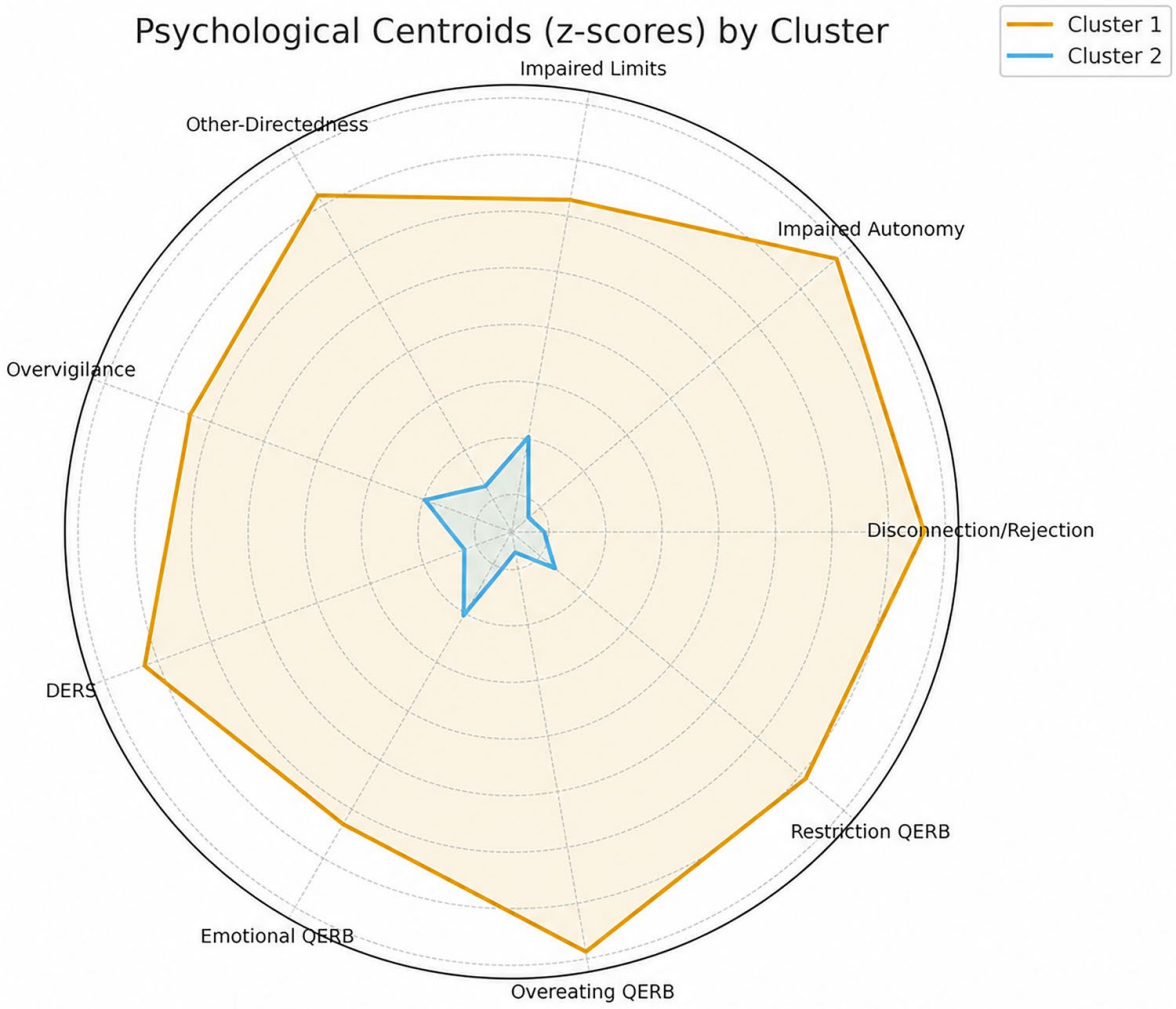
Radar plot of standardized psychological cluster profiles. Cluster 1 (high-risk profile) is characterized by higher endorsement of early maladaptive schemas (YSQ domains), greater difficulties in emotion regulation (DERS total), and elevated emotional and habitual overeating (QERB EO/HO), alongside higher restrained eating (RE). Cluster 2 (low-risk profile) displays lower scores across all psychological variables. All values are standardized *z*-scores

**Fig. 2:**
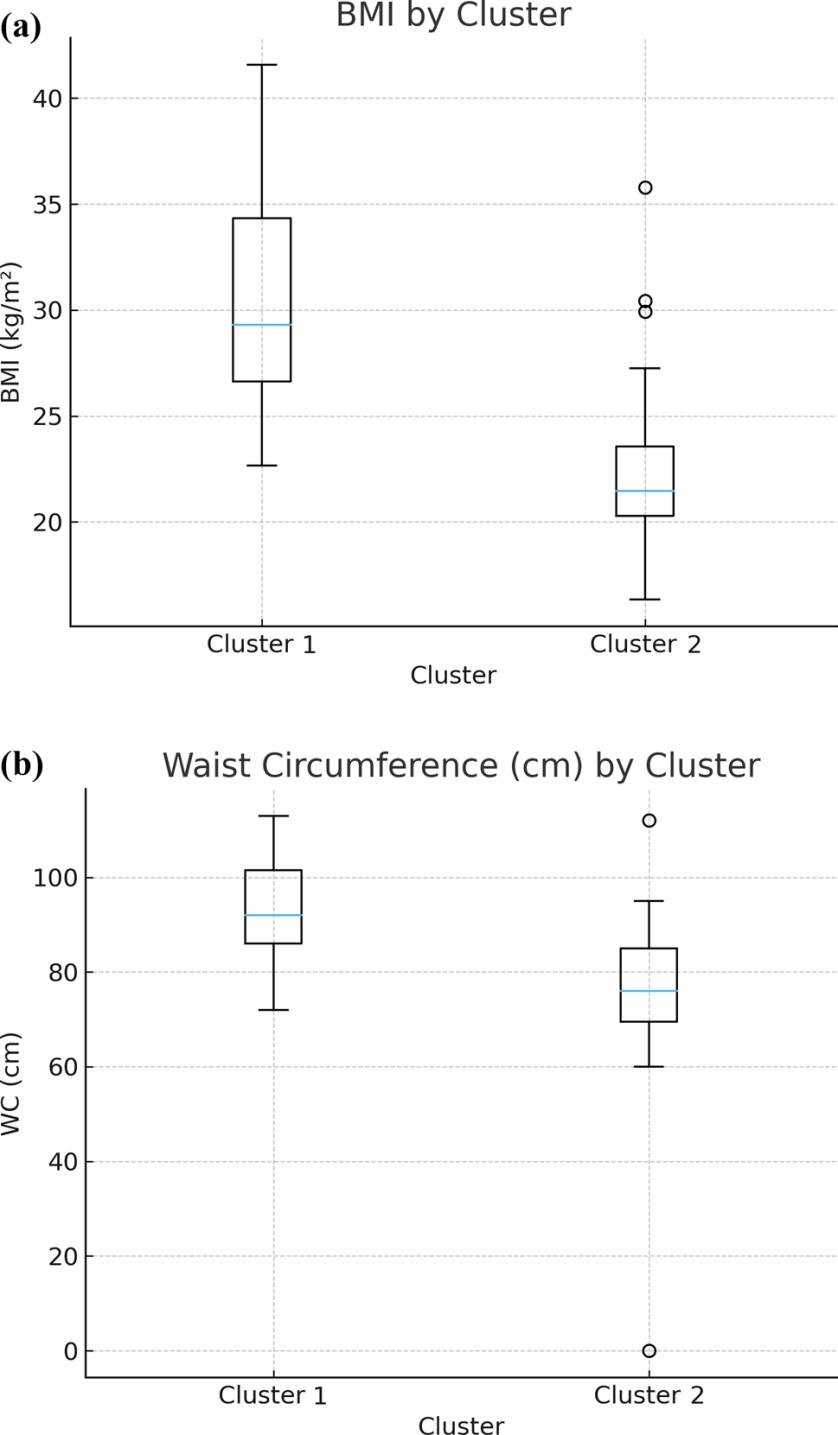
**a** Box-and-whisker plot of waist circumference (WC) by psychological cluster. Cluster 1 (high-risk profile) shows significantly higher WC compared to Cluster 2 (low-risk profile). **b** Box-and-whisker plot of body mass index (BMI) by psychological cluster. Cluster 1 (high-risk profile) shows significantly higher BMI compared to Cluster 2 (low-risk profile).

Figure 1 depicts standardized psychological centroids (radar plot). Figure 2a, b displays box-and-whisker plots for WC and BMI by cluster.

## Discussion

This pilot study examined psychological profiles of eating-related risk by clustering early maladaptive schemas, emotion regulation difficulties, and eating behaviors, with central adiposity and FTO genotype included as external validators. The findings suggest that, even within a relatively small and homogeneous sample of young women, distinct cognitive and emotional patterns may be detectable and may show associations with anthropometric indicators. These results should, however, be interpreted as exploratory and hypothesis-generating.

The two-cluster solution indicated differentiation between a higher-risk profile (Cluster 1) and a lower-risk profile (Cluster 2). Women in the higher-risk cluster reported greater endorsement of maladaptive schemas, particularly within the Disconnection/Rejection and Impaired Autonomy domains, alongside more pronounced difficulties in emotion regulation and more frequent emotional and habitual overeating. This psychological pattern co-occurred with higher waist circumference and BMI. In contrast, the lower-risk cluster showed generally lower schema activation, fewer regulatory difficulties, and less maladaptive eating behavior, together with lower anthropometric indices. Given the modest sample size and exploratory design, these differences should be interpreted cautiously and viewed as preliminary patterns rather than stable population profiles.

The observed co-occurrence of maladaptive schemas, emotion dysregulation, and overeating is consistent with prior literature. Schemas reflecting unmet attachment needs (e.g., emotional deprivation and abandonment) and impaired autonomy have been linked to emotional eating and weight-related problems [23–26]. Similarly, deficits in emotion regulation, including impulsivity and limited access to adaptive strategies, are recognized pathways to disordered eating [27, 28]. The present findings are broadly in line with these models and tentatively suggest that these vulnerabilities may cluster within individuals. However, replication in larger and more diverse samples is necessary to determine the robustness and generalizability of the identified configuration.

No significant differences in FTO rs9939609 distribution were observed between clusters. Given the limited statistical power of the present pilot sample, this null finding should not be interpreted as evidence of absence of genetic influence. It is possible that the study was underpowered to detect subtle genotype effects or interactions. Thus, the role of FTO in relation to the identified psychological patterns remains inconclusive and requires examination in larger genetically informed samples.

Overall, the findings provide preliminary support for the usefulness of a person-centered perspective when examining the co-occurrence of schema activation, emotion regulation difficulties, and maladaptive eating behaviors. At the same time, the cluster solution should be considered sample-dependent and exploratory. Future research using larger cohorts, longitudinal designs, and more heterogeneous populations will be necessary to determine whether similar psychological configurations can be reliably reproduced and whether they carry prospective clinical significance.

## Conclusion

This pilot study provides preliminary evidence that distinct patterns of schema activation, emotion regulation difficulties, and eating behaviors may be observable in young women and may show associations with anthropometric indicators of adiposity. Although FTO rs9939609 did not differentiate the identified clusters, the study was not powered to draw firm conclusions regarding genetic effects. The findings should, therefore, be interpreted as exploratory and hypothesis-generating. If replicated in larger samples, person-centered psychological profiling may contribute to a more nuanced understanding of eating-related risk processes.

### Strengths and limits

The strengths of this study include the use of a person-centered profiling approach integrating early maladaptive schemas, emotion regulation, and eating behaviors, as well as independent anthropometric validation using BMI and waist circumference. The inclusion of the FTO rs9939609 genotype as an external validator provides an additional biopsychosocial perspective.

Several limitations should be acknowledged. The cross-sectional design precludes causal inference. The sample was relatively small and demographically homogeneous (*N* = 50 young women), which limits statistical power, particularly for genetic analyses, and restricts generalizability. Cluster solutions derived from small samples may be sample-specific and require replication. Psychological variables were assessed using self-report instruments, which may introduce response bias. In addition, although inclusion criteria targeted the normal-weight and overweight/obese range, one participant presented a BMI slightly below the predefined threshold, which should be considered when interpreting descriptive parameters. Future studies with larger and more diverse samples are needed to confirm the stability and external validity of the observed patterns.

### What this study adds

This exploratory pilot study identified two psychological configurations that differed in BMI and waist circumference among young women. The person-centered approach integrating early maladaptive schemas, emotion regulation, and eating behaviors offers a preliminary framework for examining eating-related risk from a biopsychosocial perspective. These findings generate hypotheses for future research but require replication in larger samples before clinical implications can be drawn.

## Supplementary Information

Below is the link to the electronic supplementary material.

Supplementary file1 (DOCX 17 kb)

## Data Availability

Data are available from the corresponding author upon reasonable request.
