# Supplementary material for: Psychobehavioral profiles of eating risk: schema–emotion regulation patterns differ in central adiposity with genotype (FTO rs9939609) as external validator—a pilot study
Source: Eat Weight Disord. 2026 Apr 29;31(1):57. doi: 10.1007/s40519-026-01853-5 (PMC13269516; doi:10.1007/s40519-026-01853-5)
Supplement: Supplementary file 1 — Supplementary file1 (DOCX 17 kb) [file 40519_2026_1853_MOESM1_ESM.docx]

**Supplementary Table S1. Tests of normality (Shapiro–Wilk) and homogeneity of variance (Levene)**

| **Variable** | **Shapiro-W** | **p-value** | **Levene F** | **Levene p** |
| --- | --- | --- | --- | --- |
| Disconnection/Rejection | 0.975 | 0.357 | – | – |
| Impaired Autonomy | 0.951 | 0.038 | – | – |
| Impaired Limits | 0.973 | 0.312 | – | – |
| Other-Directedness | 0.970 | 0.235 | – | – |
| Overvigilance | 0.970 | 0.228 | – | – |
| DERS | 0.978 | 0.481 | – | – |
| Emotional QERB | 0.946 | 0.024 | – | – |
| Overeating QERB | 0.942 | 0.016 | – | – |
| Restriction QERB | 0.963 | 0.122 | – | – |
| WC | 0.862 | <.001 | 0.022 | 0.883 |
| BMI | 0.941 | 0.015 | 2.725 | 0.105 |

**Note.** Shapiro–Wilk tests assess departure from normality. Levene’s test was performed only for anthropometric variables (WC, BMI) across clusters. Significant deviations from normality were observed for some psychological (Impaired Autonomy, Emotional and Overeating QERB) and anthropometric variables (WC, BMI)

**Supplementary Table S2. Cluster solution fit indices for k = 2–4**

| **k** | **Silhouette** | **Calinski–Harabasz** | **BIC (approx)** |
| --- | --- | --- | --- |
| 2 | 0.196 | 11.39 | 298.22 |
| 3 | 0.161 | 8.80 | 312.69 |
| 4 | 0.145 | 7.57 | 326.34 |

**Note.** Higher silhouette and Calinski–Harabasz scores indicate better-defined clusters, while lower BIC values indicate superior model fit. The two-cluster solution showed the most favorable balance across indices and was retained for interpretation.

**Supplementary Table S3. Sensitivity analyses of FTO rs9939609 coding schemes (additive vs. dominant models)**

| **Model** | **Outcome** | **β** | **p-value** | **R²** |
| --- | --- | --- | --- | --- |
| Additive (0–1–2) | BMI_z | 0.008 | 0.965 | 0.000 |
| Additive (0–1–2) | WC_z | -0.180 | 0.337 | 0.019 |
| Dominant (AA+AT vs. TT) | BMI_z | 0.088 | 0.780 | 0.002 |
| Dominant (AA+AT vs. TT) | WC_z | -0.094 | 0.764 | 0.002 |
